# Epidemiology of Pediatric Musculoskeletal Trauma Patients Admitted to the Trauma Center of King George’s Medical University (KGMU) During COVID-19-Induced Lockdown

**DOI:** 10.7759/cureus.23648

**Published:** 2022-03-30

**Authors:** Vikas Verma, Mayank Mahendra

**Affiliations:** 1 Pediatric Orthopedics, King George's Medical University, Lucknow, IND; 2 Orthopedic Surgery, King George's Medical University, Lucknow, IND

**Keywords:** fracture in a child, covid 19, injury epidemiology, trauma pediatric, paediatric orthopedics

## Abstract

Purpose: This retrospective study aims to document the epidemiology of pediatric musculoskeletal trauma patients admitted over a one and half year period to the trauma center of King George's Medical University (KGMU) and the effect of COVID-19-induced lockdown on the timeliness of care.

Methods: We analyzed data of 174 patients for demography, types of injuries, mechanism of injuries, the site where the injury was sustained, the severity of injuries, nature of the injury, and the regions involved by the injuries.

Results: Mean age was 12.44±4.4. One hundred and twelve (67.46%) were males and 62 were females (32.54%). When compared with the period prior to lockdown, significantly higher times were recorded during the lockdown for time to a reception at the trauma center (p=0.028) and the time spent in receiving area of the trauma center (p<0.001). The most common mode of injury was low energy falls (n=68; 40.96%). The most commonly involved region was the lower limb (n=156; 51.82%). The region involved by the injury and the frequency of fracture types varied with the age of the subjects. The frequency distribution of injuries in males and females was almost similar till the age of nine years. The most common injuries of the lower extremity and upper extremities were fracture shaft of the femur and supracondylar fracture of the humerus, respectively.

Conclusion: Our study presents a precise estimate of demography and injury characteristics of pediatric musculoskeletal injuries, which may be helpful in planning and policymaking. The effect of the pandemic on the timeliness of care can be used for improving the infrastructure required to handle future waves of the pandemic.

## Introduction

Children are not small adults. They differ from adults anatomically as well as physiologically. Consequently, the types of injuries they suffer as a result of trauma are different from adults. Infants have a skeleton that is largely cartilaginous. The bones in children are softer and when a bending force is applied it results in fracture types that are different from those in adults. The most significant difference is that the bones in children have growth plates. An injury to a growth plate may lead to late deformation of the bone, necessitating further corrective surgeries. Children are more likely to suffer from intra-abdominal organ injuries as compared to adults as their skeleton being largely cartilaginous is more flexible allowing transfer of external injury to internal organs.

Management of pediatric musculoskeletal trauma is a major concern for policymakers, administrators as well as health care workers. While the epidemiology of adult musculoskeletal trauma is well researched and published, the epidemiology of pediatric musculoskeletal trauma in India has hardly been researched. A study from Norway has reported different patterns of mechanisms and resultant injuries in childhood trauma compared to adult trauma [1]. Good epidemiological data regarding pediatric musculoskeletal trauma is absolutely essential for ascertaining the resources required for managing pediatric musculoskeletal trauma at a trauma center. The objective of this study was to gather information about the patient demography, types of injuries, mechanism of injuries, the severity of the injury, nature of the injury, and the regions involved. In order to limit the spread of the pandemic, the Government of India declared a complete lockdown on March 22, 2020, which included a complete ban on air, rail, and road transport except for essential services, emergency services including emergency health services, law and order, and fire services [2]. Subsequently, the lockdown was lifted in a gradual manner starting October 1, 2020 [3]. As a secondary objective, we decided to investigate the effect of the lockdown on the timeliness of care provided to the pediatric musculoskeletal trauma patients admitted to the trauma center of King George's Medical University (KGMU).

## Materials and methods

This retrospective record review was conducted on pediatric musculoskeletal trauma patients (up to the age of 18 years) admitted to the trauma center of KGMU. The study was granted ethical approval vide letter number 2263/Ethics/2022 dated March 7, 2022. Provision of written informed consent was waived by the institutional ethics committee as the study involved a review of patient records requiring no contact with actual patients, no additional information was collected apart from what was already available in the records, and identifying information was not recorded to prevent linking with identifiable human subjects. Pediatric musculoskeletal trauma victims up to the age of eighteen years admitted over a period of six months starting October 1, 2019 to March 31, 2021 were included in the study. We did not include patients with incomplete records.

Information regarding the timeliness of care was recorded by recording the time spent since the injury to arrival to the receiving area (casualty or holding area) in the trauma center, time spent by the patient in receiving area (casualty or holding area) before being transferred to the concerned department for definitive care. Patients or their attendants were asked for time spent since the injury to arrival to the receiving area. Once a patient reaches the receiving area of KGMU, a prescription paper is generated on which the time at which the patient was received is recorded. When the patient reaches the concerned department for definitive care, an admission slip is generated on which the time of admission to the concerned department is recorded. These were used to calculate the time spent in receiving area before admission for definitive care. The injury was coded using the Abbreviated injury scale which was used to calculate the Injury Severity Score (ISS). Injuries were recorded using the patient records including the case sheet, X-rays, and any other investigations. The severity of the injury was recorded as the ISS. The clinical type of fracture was recorded as simple or compound. The type of injury was recorded as blunt or penetrating or a combination of the two. Anatomical regions involved, mode of injury, and the site where the injury was sustained were recorded. Modes of injury were recorded as road traffic injury, low energy fall, high energy fall, direct blow, sports-related, occupation-related, domestic accident, and deliberate self-harm. The nature of musculoskeletal injury was recorded as fracture, fracture dislocation, crush injury, traumatic amputation, dislocation, physeal injury, or a combination of more than one type. The type of admission was recorded as direct or referred from some other center.

Abbreviated injury scale code is an anatomical injury severity scoring system that classifies an individual injury by body region according to a severity scale of 0-6 [4]. It is the basis of calculating ISS [5] in an injured patient. In literature low energy falls have been described as those in which the victim fell from a height of less than 20 feet [6]. The same was used by us to classify falls into low energy or high energy. Outcome of admission was recorded as discharged, left against medical advice, absconded, expired or transferred to some other department.

The data collected was tabulated using Microsoft Excel software and analyzed using SPSS v 21.0 version for Windows (IBM, Armonk, NY, USA). Continuous data was described using means and standard deviation. Categorical data was summarized using frequency distribution tables and chi-square test. Unpaired T-test was used to compare the means of continuous variables (time spent before a reception at KGMU; and time spent in receiving area) in the pre-lockdown, lockdown, and post-lockdown periods.

## Results

One hundred and seventy-four pediatric musculoskeletal trauma patients meeting the inclusion criteria were admitted during the study period. Eight patients with incomplete records were excluded from the study. Therefore, the results of 166 patients are described. One hundred and twelve (67.4%) were males and 54 (32.6%) were females; the average age of the patients was 12.44±4.4. One hundred and twelve (67.46%) were males and 62 were females (32.54%). One hundred and forty-three patients (86.14%) were admitted after being referred from some other center.

Seventy-eight (46.98%) were admitted in the period prior to lockdown, 33 (19.88%) were admitted during the lockdown and 55 (33.13%) were admitted in the period after the lockdown. Time spent before the patient was received in the trauma center was 52.54±76.85 hr in the period before the lockdown, 90.03±90.63 hr during the lockdown, and 71.18±51.53 hr after the lockdown. Meantime spent before the patient was received at KGMU was significantly higher in the lockdown compared to the period before the lockdown (p=0.028). Meantime spent before the patient was received at KGMU was not found to be significantly different between pre-lockdown and post lockdown (p=0.12). Meantime spent before definitive care was provided to the patient was 1.05±0.31 hr prior to lockdown, 15.02±7.08 during the lockdown, and 17.45±2.13 hr after the lockdown. Meantime spent at the receiving area was significantly higher in the lockdown compared to the period before lockdown (p<0.0001) as well the period after the lockdown (p<0.0001).

**Figure 1:**
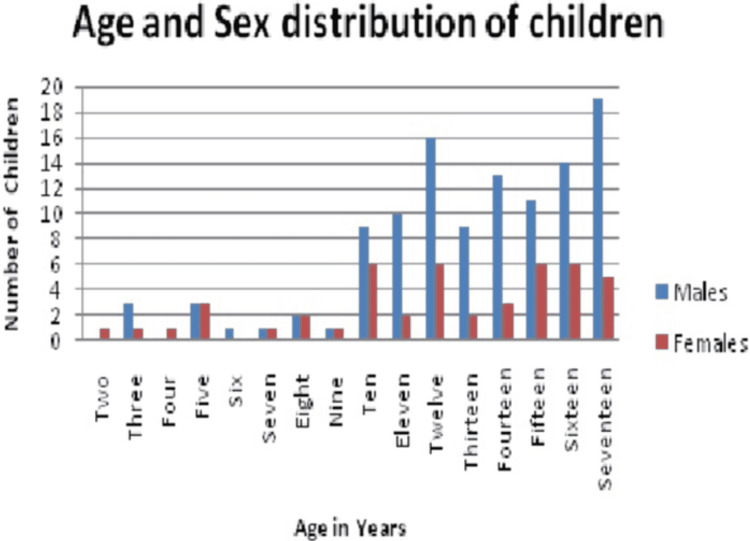
The age-wise frequency of musculoskeletal injuries in males and females

Mean ISS was 7.03±3.93 prior to lockdown, 12.58±7.62 during the lockdown, and 9.24±6.44 after the lockdown. Means ISS was significantly higher in the lockdown compared to the period before the lockdown (p<0.0001) as the period after the lockdown (p=0.029). The frequency distribution of injuries in males and females was almost similar till the age of nine years. After the age of nine years, males had a higher frequency of injuries compared to females (Figure 1).

The most common mode of injury was low energy falls (n=68; 40.96%). Age-wise distribution of mechanism of injury is shown in Table 1.

**Table 1:** Age-wise mechanism of injury

Age	Mechanism of Injury	N	%
0-2 years	Low energy fall	08	80.00
	Road traffic accident	01	10.00
	High energy fall	00	00.00
	Others	01	10.00
3-6 years	Low energy fall	09	81.82
	Road traffic accident	02	18.18
	High energy fall	00	0.00
	Others	00	0.00
7-12 years	Low energy fall	30	56.60
	Road traffic accidents	05	0.9.43
	High energy fall	16	30.19
	Others	02	03.77
13-17 years	Low energy falls	30	37.03
	Road traffic accidents	30	37.03
	High energy fall	26	32.09
	Others	05	06.17

One hundred and fifty-five patients had pure musculoskeletal injuries (93.37%). One hundred and thirty-eight patients had a fracture (83.13%). Of these, 131 patients had a fracture, six had a fracture dislocation and one had a simple fracture of femur combined with crush injury of the ipsilateral foot. One hundred and ten patients had a simple fracture, 28 had a compound fracture and eight had a combination of simple and compound fractures. The lower limb was the most commonly involved region (n=70; 42.17%) (Table 2).

**Table 2:** Injury characteristics

Variable		No	%
Type of injury	Blunt	155	93.37
	Penetrating	1	0.60
Nature of injury	Fracture	131	78.92
	Crush	1	0.60
	Amputation	4	2.41
	Dislocation	1	0.60
	Physeal injury	2	1.20
	Fracture dislocation	6	3.61
	Combination	21	12.65
Clinical type of fracture	Simple	110	66.27
	Compound	28	16.87
	Combination	8	4.82
Region involved	Upper Limb	59	35.54
	Lower Limb	70	42.17
	Pelvis	2	1.20
	Spine	13	7.83
	Pelvis with extremity	7	4.22
	Spine with extremity	8	4.82
	Both extremities	7	4.22

**Figure 2:**
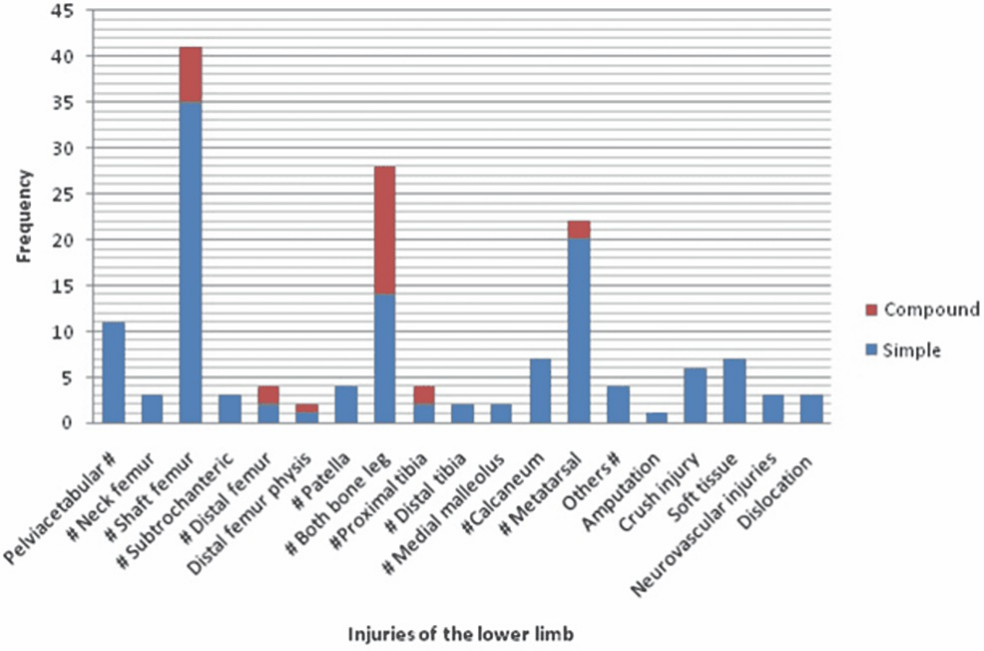
Distribution of injuries in the lower limb

A total of 295 injuries were recorded in 166 patients. Two hundred and fifty five of these were fractures, eight were crush injuries, six were traumatic amputations, three were arterial injuries, six were nerve injuries, four were dislocations, eight were lacerations, four were de-gloving injuries, and one patient had a brachial plexus injury. The most commonly involved region was the lower limb (n=156; 51.82%). The two most common injury of the lower limb was fracture of shaft of femur and fracture of both bone leg (Figure 2).

**Figure 3:**
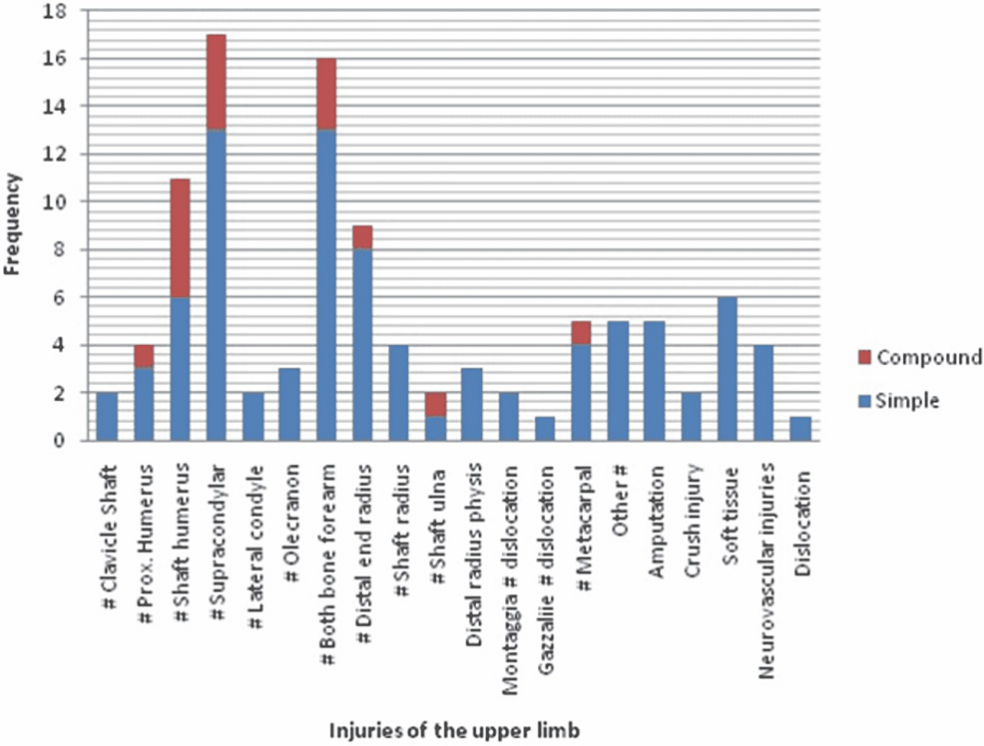
Distribution of injuries in the upper extremity

One hundred and four injures (34.55%) were recorded in upper extremity. The two most common injuries in the upper limb were supracondylar fracture of humerus and fracture both bone forearm (Figure 3). Spinal injuries (n=29 fractures) constituted 9.63% of all injuries. Eleven non-orthopedic injuries were recorded.

Up to the age of two years, the most common fracture was distal end radius physeal injury (30%) followed by fracture of the shaft of clavicle (20%). In the age group 3-6 years, the most common injury was fractures of both bones of forearm (31.25%), followed by fractures of both bone leg (25%). In the 7-12 years age group, the most common fracture was of shaft of femur (29.54%) followed by fracture of supracondylar humerus (20.45%). In the 13-17 years age group, the most common fracture type was that of shaft of femur (15.76%) followed by a fracture to the spine (13.58%) (Table 3). Age-wise sex ratio and limb distribution is shown in Table 3.

**Table 3:** Age distribution of subjects according to sex ratio, limb distribution, and common fracture types

Age group (number of fractures)	Male%: Female%	Upper limb%: Lower limb%	Two most common fractures	N(% age)
0-2 (10)	40:60	80:20	Distal radius physeal injury	3(30.00)
			Fracture of clavicle	2(20.00)
3-6 (16)	53.84: 46.14	56.25:43.75	Both bone forearm	5(31.25)
			Both bone leg	4(25.00)
7-12 (44)	68.25:31.75	56.10: 43.90	Shaft femur	13(29.54)
			Supracondylar humerus	9(20.45)
13- 17 (184)	69.47: 30.53	73.02:26.98	Fracture shaft femur	29(15.76)
			Vertebral fracture	25(13.58)

One hundred and fifty-two patients (91.56%) were discharged; seven (4.21%) left the hospital against medical advice (LAMA); four patients (2.41%) absconded from the hospital and three patients (1.81%) were transferred to plastic surgery for flap coverage. None of the patients died.

## Discussion

In the present study, 67.4% of the enrolled subjects were males, which is about twice the percentage of females enrolled. In our study, 81.48% (n=44) of the female patients (n=44) had a pure orthopedic injury and 91.07% (n=102) of the males had a pure orthopedic injury. Naranjie et al. [7], Lyons et al. [8], and Wolfe et al. [9] have also reported a higher incidence of injuries in male children compared to females. Naranjie et al. [7] using population-based estimates have reported an 18% higher incidence of fractures in male children compared to similar-aged females. Lyons et al. [8] using population-based estimates have reported a 12% higher incidence of fractures in male children compared to female children. Hedstrom et al. have reported that a higher incidence of fractures in males is attributed to greater participation in sports in males as well as a greater tendency of males to indulge in risk-taking behavior [10]. However, a study by Baig et al. that controlled for sports participation [11] reported that male athletes were twice more likely than females to sustain a fracture. The present study found an almost similar frequency of fractures till the age of nine years in both sexes. However, after the age of nine years, there was a higher frequency of injuries in males compared to females.

The distribution of musculoskeletal injuries in the upper extremity and lower extremity varies with age. Wolfe et al. have reported that before children start walking, fractures tend to be more common in the upper extremity [9]. Once a child starts walking and running, fractures tend to be more common in the lower extremity [9,11]. In the present study, we have also found a similar pattern. In the age group of children up to two years of age, distal radial physeal injuries were the most common injuries. In the 3-6 years age group, both bone fractures of the forearm were the most common fractures. However, the second most common fracture in this age group was fracture of both bone legs, which probably reflects the increased ambulatory status of children. In the 7-12 years age group, the most common fracture was fracture of shaft of the femur, and the second most common fracture was supracondylar fracture of the humerus. In the 13-17 years age group the most common fracture was fracture shaft of the femur and the second most common fracture was a fracture to the vertebral column. A high prevalence of spinal injuries in our data set can be explained by a high number of referred cases in our study. A higher number of referred cases could be the effect of COVID-19-induced lockdown as private healthcare setups not providing emergency services were not admitting patients during the lockdown.

The most common mechanism of injury reported in the present study is low energy falls. Maryanpaa et al. have also reported fall to be the most common cause of pediatric fractures [12]. The mechanism of injury varies with age [11-13]. In the present study, we have also found a similar pattern. In our study, low energy falls were the most common mechanism of injury in the age groups <2 years, 3-6 years, and 7-12 years. However, in the age group 13-17 years, the most common mechanism of injury in our study were road traffic accidents and low energy falls. However, in this age group, high energy falls were also quite common.

This study has reported a significant increase in the time spent before the patient was received at KGMU during lockdown. This is on expected lines as the lockdown resulted in a major disruption of traffic [14]. A concerning finding of this study is the rise in time spent in receiving area before transfer for definitive care. This seems to be a result of limited COVID-19 testing facilities combined with mandatory RTPCR for COVID-19 before transfer for definitive care. In early February, there were only 14 laboratories in India which could test do an RTPCR for COVID-19 [14]. This number increased to 106 by the end of March [15]. The virology lab of KGMU conducted RTPCR test on samples received from other parts of KGMU in addition to the patients being admitted at KGMU. Thus an overloading of the facility might have been the reason for an increase in the time spent in receiving area. A concerning finding of our study is that once the lockdown was lifted, there was an increase in the time spent in receiving (15.02±7.08 hr to 17.45±2.13 hr) area. A probable explanation for this increase is that in the period after the lockdown, elective admissions were allowed resulting in a greater number of admissions which translated into a higher number of RTPCR tests and an even greater time spent in holding area.

## Conclusions

The present study presents a precise estimate of pediatric musculoskeletal injuries distributed by age, gender, mechanism of injury, and region-wise distribution of injuries. This information can help in the policymaking and planning of the nascent trauma care system of India. Pediatric trauma management is not a part of the graduate or post-graduate curriculum of medical studies in India. The results of our study may be used to improve the training and teaching of the resource personnel involved in pediatric musculoskeletal trauma care. The method used by us to generate baseline epidemiological data of musculoskeletal injuries may be used as a model to build better methods to generate epidemiological data of pediatric injuries involving other regions. The effect of the pandemic on the timeliness of care has been documented by this study which can be used for improving the infrastructure required to handle future waves of the pandemic.

## References

[1] Nesje E, Valøy NN, Krüger AJ, Uleberg O (2019). Epidemiology of paediatric trauma in Norway: a single-trauma centre observational study. Int J Emerg Med.

[2] (2021). Guidelines on the measures to be taken by Ministries/Departments of Government of India, State/Union Territory governments and State/Union Territory authorities for containment of Covid epidemic in the country. https://www.mha.gov.in/sites/default/files/Guidelines_0.pdf.

[3] (2020). Guidelines for reopening [As per Ministry of Home affairs order No. 40-3/2020-DM-I (A) dated 30th September, 2020]. https://www.mha.gov.in/sites/default/files/MHAOrderDt_30092020.pdf.

[4] (2022). Abbreviated injury scale. Association for advancement of automotive medicine. https://www.aaam.org/abbreviated-injury-scale-ais/.

[5] Baker SP, O'Neill B, Haddon W Jr, Long WB (1974). The injury severity score: a method for describing patients with multiple injuries and evaluating emergency care. J Trauma.

[6] Helling TS, Watkins M, Evans LL, Nelson PW, Shook JW, Van Way CW (1999). Low falls: an underappreciated mechanism of injury. J Trauma.

[7] Naranje SM, Erali RA, Warner WC Jr, Sawyer JR, Kelly DM (2016). Epidemiology of pediatric fractures presenting to emergency departments in the United States. J Pediatr Orthop.

[8] Lyons RA, Delahunty AM, Kraus D, Heaven M, McCabe M, Allen H, Nash P (1999). Children's fractures: a population based study. Inj Prev.

[9] Wolfe JA, Wolfe H, Banaag A, Tintle S, Koehlmoos TP (2019). Early pediatric fractures in a universally insured population within the United States. BMC Pediatr.

[10] Hedström EM, Svensson O, Bergström U, Michno P (2010). Epidemiology of fractures in children and adolescents. Acta Orthop.

[11] Baig M A review of epidemiological distribution of different types of fractures in paediatric age. Cureus.

[12] Maryanpaa MK, Maketie O, Kallio PE (2010). Decreasing incidence and changing pattern of childhood fractures: a population based study. J Bone Mineral Res.

[13] Chaudhary S, Figueroa J, Shaikh S (2018). Pediatric falls ages 0-4: understanding demographics, mechanisms, and injury severities. Inj Epidemiol.

[14] Mampatta SP (2020). Covid-19 impact: 60% decline in city traffic as restrictions take effect. March.

[15] (2022). How India scaled up its laboratory testing capacity for COVID 19. https://www.who.int/india/news/feature-stories/detail/how-india-scaled-up-its-laboratory-testing-capacity-for-covid19.

